# Single cell RNA-seq data clustering using TF-IDF based methods

**DOI:** 10.1186/s12864-018-4922-4

**Published:** 2018-08-13

**Authors:** Marmar Moussa, Ion I. Măndoiu

**Affiliations:** University of Connecticut, Storrs, 06269 CT USA

**Keywords:** Single cell RNA-Seq, Clustering, TF-IDF

## Abstract

**Background:**

Single cell transcriptomics is critical for understanding cellular heterogeneity and identification of novel cell types. Leveraging the recent advances in single cell RNA sequencing (scRNA-Seq) technology requires novel unsupervised clustering algorithms that are robust to high levels of technical and biological noise and scale to datasets of millions of cells.

**Results:**

We present novel computational approaches for clustering scRNA-seq data based on the Term Frequency - Inverse Document Frequency (TF-IDF) transformation that has been successfully used in the field of text analysis.

**Conclusions:**

Empirical experimental results show that TF-IDF methods consistently outperform commonly used scRNA-Seq clustering approaches.

## Background

The recent advances in single cell RNA sequencing (scRNA-Seq) technologies promise to unveil novel cell types and uncover subtle regulatory processes that are undetectable by analyzing bulk samples. Currently, droplet-based technologies such as the Chromium Megacell commercialized by 10x Genomics can quickly and inexpensively generate scRNA-Seq expression profiles for up to millions of cells. Indeed, a dataset recently made public by 10x Genomics is comprised of 1.3 million mouse brain cells. However, the sequencing depth of each cell in such datasets is typically very low, resulting in many missing gene expression levels (the above 10x dataset has a mean of only 23,185 reads per cell, with a median of only 1927 genes detected per cell). The large amounts of data and high levels of noise render many unsupervised clustering methods developed for bulk gene expression data [1] unusable, prompting the development of a new generation of clustering tools.

In this paper, we propose several computational approaches for clustering scRNA-Seq data based on the *Term Frequency - Inverse Document Frequency (TF-IDF)* transformation commonly used for text/document analysis. Empirical evaluation on simulated and real cell mixtures of FACS sorted cells with different levels of complexity suggests that the TF-IDF methods consistently outperform existing scRNA-Seq clustering methods. In the Methods section we detail several commonly used scRNA-Seq clustering methods, provide background on the TF-IDF transformation and its proposed application to scRNA-Seq data clustering, and describe the experimental setup and accuracy metrics used in our empirical assessment. In the Results section we present the results of a comprehensive evaluation comparing the accuracy of the proposed TF-IDF based methods with that of existing methods on cell mixtures with both simulated and real proportions. Finally, in the Conclusions section we outline directions for future work.

## Methods

**Fig. 1:**
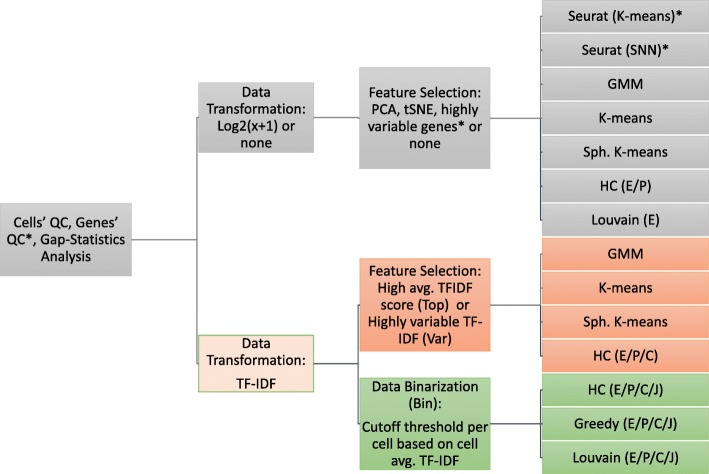
Compared scRNA-Seq clustering methods. *For Seurat, QC and gene selection were carried out as suggested in [4]

We did a preliminary assessment of twelve previously proposed methods for clustering scRNA-Seq data, and selected for the final assessment nine methods that had consistently high accuracy as described in the Results section. Our assessment also did a preliminary analysis of twenty four methods based on the TF-IDF transformation, out of which we selected nineteen methods for inclusion in the final comparison. A summary of the compared methods is given in Fig. 1. We next describe the common data processing employed for all methods, then give details of individual methods.

Synthetic datasets comprised of two to seven cell types mixed in different proportions were generated as described below using 3^′^-end scRNA-Seq data generated using the 10x Genomics platform from FACS sorted immune cells [2]. For experiments on these mixtures all methods take as input the raw *Unique Molecular Identifier (UMI)* counts generated using 10x Genomics’ CellRanger pipeline for each gene and cell as described in [2]. Using UMI counts rather than read counts reduces bias introduced by PCR amplification in scRNA-Seq protocols. For all 10x Genomics datasets we first filtered the cells based on the number of detected genes and the total UMI count per cell [3]. We also removed outliers based on the median-absolute-deviation (MAD) of cell distances from the centroid of the corresponding cell type. We also performed basic gene quality control by applying a cutoff on the minimum total UMI count per gene across all cells and removing outliers based on MAD. For Seurat [4], the cell and gene quality control was performed as recommended by the authors and described below.

A second test dataset consisted of scRNA-seq data generated using the Smart-seq2 protocol from seven types of pancreatic cells [5]. For this dataset clustering was performed twice, once using *Reads Per Kilobase per Million (RPKM)* estimates and once using raw read counts reported in [5]. No cell QC was performed for this set. The same gene QC as described above for 10x UMI data was performed; again for Seurat, the recommended defaults for gene quality control and selection were applied.

**Fig. 2:**
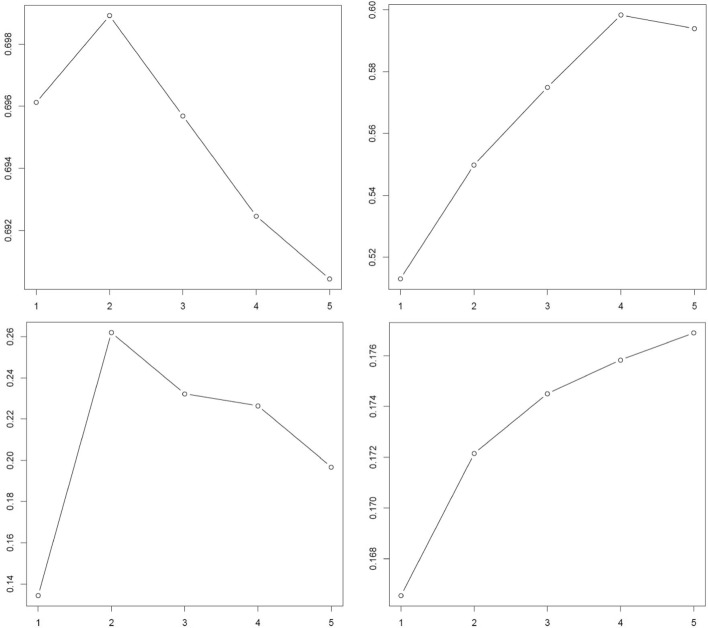
Clockwise from top left: gap statistics for log-transformed, log-transformed PCA, tSNE, and TF-IDF transformed and binarized expression levels of a 7:1 mixture of regulatory_t and naive_t cells. The x-axis gives the number of clusters K and the y-axis gives the gap statistic in (1)

For all methods, we determine an ‘optimal’ number of clusters using the gap statistic approach introduced in [6]. Briefly, the optimal number of clusters is selected as argmax_*k*_Gap_*n*_(*k*), where the gap statistic for clustering *n* points into *k* clusters is given by

1 $$ \text{Gap}_{n}(k) = E_{n}^{*}\{\log W_{k}\} - \log W_{k},  $$

i.e., the difference between the logarithm of the normalized sum *W*_*k*_ of pairwise distances in the *k* clusters and its expectation under a null reference distribution generated by Monte Carlo sampling. The gap statistic analysis was independently performed for each transformation applied to the data (log-transform, PCA, tSNE, TF-IDF, etc.) as the gap statistics, and hence the optimal number of clusters, are sensitive to these transformations (Fig. 2). The gap statistic based estimate was used to directly specify the number of clusters for all methods except *Seurat, Seurat_SNN* and graph-based clustering algorithms, which determine the number of clusters internally.

When the number of clusters determined by *Seurat_SNN* and graph-based clustering algorithms was lower than the gap statistic estimate additional partitioning steps were performed as described below to enforce a minimum number of clusters.

### Existing scRNA-Seq clustering methods

We included in our comparison several commonly used methods. First, we included two methods from the Seurat package [4], one based on K-means and one based on graph clustering. Following the Granatum pipeline [7], we included K-means and hierarchical clustering with Euclidean and Pearson distances based on a 2-dimensional projection of the data using the *t-distributed Stochastic Neighbor Embedding (tSNE)* transformation [8]. Also from Granatum, we tested K-means using the log2(x+1) transformed data. Using the log2(x+1) transform of the data followed by PCA, we tested a Gaussian mixture model (GMM) based algorithm, a K-means algorithm similar to that implemented in the CellRanger pipeline distributed by 10x Genomics [9], as well as spherical K-means and hierarchical clustering algorithms, again with both Euclidean and Pearson correlation distances. Finally, similar to the graph-based algorithms implemented in the latest version of the CellRanger pipeline [9], we tested the graph-based Louvain clustering algorithm [10] with Euclidean distance over log2(x+1) transformed data. Details on individual methods are as follows.

#### Seurat, Seurat_SNN

To test Seurat, we followed the guided clustering workflow recommended in the tutorial at [11] by first applying the recommended cell quality filtering based on the number of detected genes, minimum 200 per cell, and percentage of reads from mitochondrial genes. Then, as recommended by Seurat’s authors, we ‘regressed out’ uninteresting sources of variation such as technical noise and batch effects. As suggested in [12], regressing out these effects improves downstream dimensionality reduction and clustering. We then used Seurat’s MeanVarPlot() with its default values to identify genes that are outliers on the ‘mean variability plot’ as recommended by Seurat’s authors. After selecting highly variable genes and performing PCA analysis, we used Seurat’s DOKMeans() function which performs K-means clustering on both genes and cells; we refer to this method as *Seurat* in the Results section. We also used the FindClusters() function which uses the top principal components and identifies clusters of cells by a shared nearest neighbor (SNN) modularity optimization based clustering algorithm that first calculates k-nearest neighbors and constructs the SNN graph, then optimizes the modularity function to determine clusters; this method is referred to as *Seurat_SNN*.

#### Gaussian mixture model based clustering (Log_PCA_GMM)

We used the mclust R package [13] to perform clustering by fitting a finite Gaussian Mixture Model (GMM) using expectation-maximization. We first performed Principal Component Analysis (PCA) of the log2(x+1) transformed UMI count matrix and ran mclust on the top 10 principal components.

#### K-means clustering variants (Log_Kmeans, Log_PCA_Kmeans, tSNE_Kmeans)

K-means clustering [14] aims to partition *n* points (cells in our case) into *k* clusters such that the total intra-cluster variance is minimized. Motivated by the similar clustering option provided in the Granatum pipeline from [7] we included in the comparison a K-means variant (called *Log_Kmeans*) that takes as input the log2(x+1) transformed UMI counts. We also followed an approach similar to that adopted in the CellRanger pipeline distributed by 10x Genomics [9], referred to as *Log_PCA_Kmeans*, in which the PCA is run on the log2(x+1) transformed UMI counts and K-means clustering is performed on the first 10 principal components. Finally, and again motivated by the Granatum pipeline from [7], we included a K-means variant run on the 2-dimensional tSNE transformation of the data (*tSNE_Kmeans*).

#### Spherical K-means with log transform and PCA (Log_PCA_sKmeans)

In this method we used the spherical K-means algorithm [15] to cluster the log2(x+1) and PCA transformed data. Instead of Euclidean distance, spherical K-means employs the *cosine dissimilarity*,

2 $$ 1-\cos(\theta) =1 - {\frac {\sum\limits_{i=1}^{n}{A_{i}B_{i}}}{{\sqrt {\sum\limits_{i=1}^{n}{A_{i}^{2}}}}{\sqrt {\sum\limits_{i=1}^{n}{B_{i}^{2}}}}}}  $$

based on the angle between two feature vectors *A* and *B*, which has been shown to be more robust to large differences in total vector weights. We added this method here as we wanted to compare its performance with the spherical K-means applied to TF-IDF transformed data described in next subsection.

#### Hierarchical clustering variants (Log_PCA_HC_E, Log_PCA_HC_P, tSNE_HC_E, tSNE_HC_P)

Agglomerative hierarchical clustering is a “bottom up” approach: each observation starts in its own cluster, and pairs of clusters are iteratively merged based on inter-cluster distances. Ward’s method [16] was used as linkage criterion. We included in the comparison four variants of hierarchical clustering, in which the algorithm was run using Euclidean and Pearson correlation distances on either the first 10 principal components of the log2(x+1) UMI counts (methods referred to as *Log_PCA_HC_E* and *Log_PCA_HC_P*, respectively), or on the 2-dimensional tSNE transformation of the data as in [7] (*tSNE_HC_E* and *tSNE_HC_P*).

#### Graph based Louvain clustering algorithm (Log_Louvain_E)

We also included in our comparison a graph-based Louvain clustering algorithm similar to that provided by the current version of the CellRanger pipeline distributed by 10x Genomics [9]. This method takes as input the log2(x+1) transformed UMI counts and builds a graph by connecting pairs of cells with Euclidean pairwise distance above a certain threshold. For our experiments we scaled the distance values to the range 0 to 1 and set a cutoff of 0.01 to build a rather dense but weighted graph. We then apply the Louvain for modularity optimization [10] as implemented in igraph R [17] package to identify communities (clusters) of cells.

Different from our method, the CellRanger pipeline implements Louvain modularity optimization on a sparse nearest-neighbor graph, where each cell is linked to its k nearest Euclidean neighbors, where k is set to scale logarithmically with the number of cells. CellRanger’s implementation also includes an additional cluster-merging step which consists of hierarchical clustering on the cluster-medoids in PCA space followed by merging of sibling clusters with no differentially expressed genes at an FDR of 0.05; such a step was not included in our implementation.

### TF-IDF scoring

TF-IDF, which stands for *Term Frequency times Inverse Document Frequency*, is a data transformation and a scoring scheme typically used in text analyses for measuring whether or not and how concentrated into relatively few documents the occurrences of a given word are [18]. Given a collection of *N* documents, let *f*_*ij*_ be the number of occurrences of word *i* in document *j*. The *term frequency* of word *i* in document *j*, denoted by *TF*_*ij*_, is defined as

3 $$ TF_{ij} = f_{ij} / \max_{k} f_{kj}  $$

Here, the term frequency of word *i* in document *j* is the number of occurrences normalized by dividing it by the maximum number of occurrences of any word in the same document, sometimes this is done after excluding stop words. The normalization is needed to make it possible to compare term frequencies for documents of different lengths. After normalization, the most frequent word in a document always gets a term frequency value of 1, while other words get fractional values as their respective term frequencies. The *Inverse Document Frequency* of word *i* is defined as

4 $$ IDF_{i} = \log_{2} (N/n_{i}).  $$

where *n*_*i*_ denotes the number documents that contain word *i* among the *N* documents in the collection. Finally, the *TF-IDF score* for word *i* in document *j* is defined to be *TF*_*ij*_×*IDF*_*i*_. Words with the highest TF-IDF score in a document are often the terms that best characterize the topic of that document.

To apply TF-IDF scores for scRNA-Seq data we consider the cells to be analogous to documents; in this analogy, genes correspond to words and UMI counts replace word counts. The TF-IDF scores can then be computed from UMI counts using Eqs. (3) and (4). Similar to document analysis, the genes with highest TF-IDF scores in a cell are expected to provide most information about the cell’s type.

We explored two different approaches of using TF-IDF scores for scRNA-Seq clustering. In first approach TF-IDF scores were used to select a subset of the most informative genes that were then used for performing clustering. In the second approach all genes are used for clustering but the gene expression data was first binarized based on a TF-IDF cutoff. Each of these data transformations were combined with a number of clustering algorithms, as detailed in the following two subsections.

### scRNA-Seq clustering based on TF-IDF gene selection

We tested two alternatives methods for TF-IDF based gene selection: using the genes with highest TF-IDF average and using the genes with highest variability in TF-IDF values.

In the first method, referred to as *Top*, we fitted a 2-mixture GMM model to the distribution of TF-IDF gene averages using mclust, and selected the genes assigned to the mixture component with highest mean. In case this resulted in a list of more than 3000 genes, we retained only the top 3000 genes when ranking the genes based on the number cells in which they are detected.

**Fig. 3:**
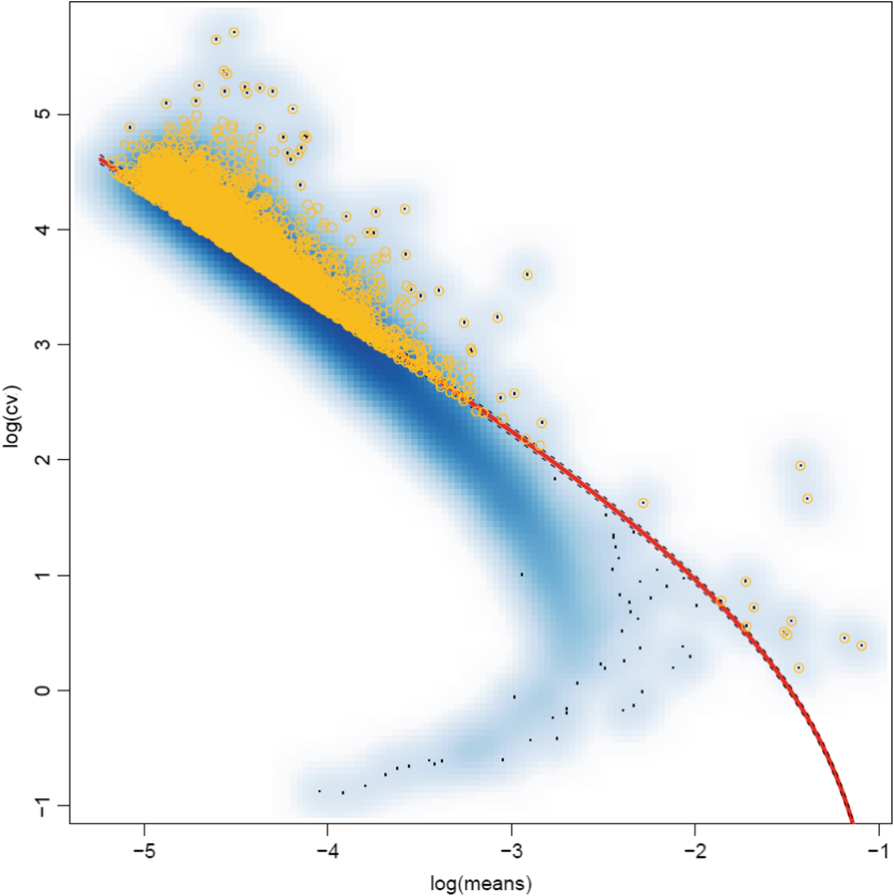
Highly variable genes for a 1:1 mixture of b_cells and cd14_monocytes

In the second method, referred to as *Var*, we identified genes with high TF-IDF variability by analyzing the relationship between the coefficient of variation (CV) and average expression levels as described in [19]. We first computed for each gene the sample TF-IDF mean and coefficient of variation *CV*, which is a standardized measure of dispersion. We then fitted a regression line for the observed pairs of mean/*CV* values (plotted on log-log scale in Fig. 3). Finally, we computed for each gene the difference between the observed *CV* and the *CV* expected for the observed mean based on the regression line, and retained for clustering analysis only the top 30% of the genes ranked by this difference (shown in yellow in Fig. 3).

After applying the TF-IDF transform to the UMI count matrix and performing gene selection using the above two methods, clustering was performed using one of the following algorithms: *Gaussian mixture model based clustering (TF-IDF_Top_GMM, TF-IDF_Var_GMM).* The expectation-maximization clustering algorithm implemented in the mclust R package [13] was applied to the TF-IDF scores of genes selected using the *Top*, respectively *Var* methods. *K-means (TF-IDF_Top_Kmeans, TF-IDF_Var_Kmeans).* Similarly, we applied K-means clustering to the TF-IDF scores of genes selected using either *Top* or *Var*. *Spherical K-means (TF-IDF_Top_sKmeans, TF-IDF_Var_sKmeans).* We also used the spherical K-means algorithm [15] on TF-IDF scores of genes selected using *Top*, respectively *Var*. *Hierarchical clustering (TF-IDF_Top_HC_E, TF-IDF_Top_HC_P, TF-IDF_Top_HC_C, TF-IDF_Var_HC_E, TF-IDF_Var_HC_P, TF-IDF_Var_HC_C).* Finally, we performed hierarchical clustering with Ward aggregation on the TF-IDF scores of selected genes using Euclidean, Pearson correlation, as well as cosine distance (2) – the latter metric was included as it is often employed in conjunction with TF-IDF for text analysis [20].

### scRNA-Seq clustering using TF-IDF based binarization

**Fig. 4:**
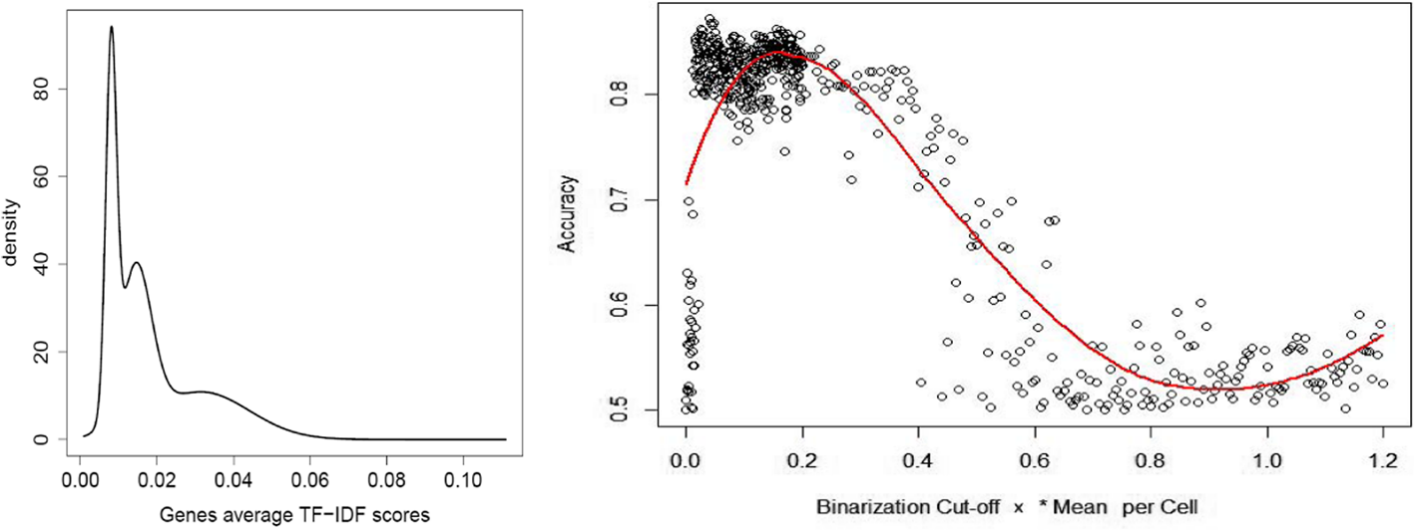
Left: Distribution of TF-IDF gene averages for a 1:1 mixture of memory and regulatory T cells. Right: Binarization cutoff effect on macro accuracy of TF-IDF_Bin_HC_C method on the same cell mixture

The distribution of mean TF-IDF scores of the genes (plotted for a mix of 1,000 memory and 1,000 regulatory T cells in the left panel of Fig. 4) typically exhibits a long tail. The genes with very high mean TF-IDF scores are potentially the most informative in identifying the underlying cell types. The final group of TF-IDF based methods uses this intuition by binarizing the gene expression data. We first selected a suitable TF-IDF cutoff and then, for each cell, we set the *expression signature* of all genes with a TF-IDF above the cutoff to 1, and all remaining signatures to 0. Cells sharing the same type are expected to have highly similar 0/1 expression signature vectors. By setting to 1 only the ‘informative’ genes in each cell we aim to remove unnecessary noise and achieve better clustering accuracy. Although the choice of TF-IDF cutoff can affect the clustering accuracy, as shown in the right side of Fig. 4) for a sample cell mixture, near maximum accuracy is achieved by using a cutoff value equal to 0.1 × the mean of the per-cell non-zero TF-IDF values. All experimental results presented in the Results section are based on this cutoff.

The resulting binary expression signatures were then clustered using one of the following algorithms: *Hierarchical clustering with Euclidean, Pearson, cosine and Jaccard distances (TF-IDF_Bin_HC_E, TF-IDF_Bin_HC_P, TF-IDF_Bin_HC_C, TF-IDF_Bin_HC_J).* Hierarchical clustering with Ward aggregation was applied to the binarized TF-IDF expression signature vectors using Euclidean, Pearson correlation, and cosine distances (2), respectively, to compare with the previous variations of hierarchical clustering based on the same distances. Additionally, we performed hierarchical clustering with Ward aggregation using the *Jaccard distance* to measure dissimilarity between cells. This is defined as 1 - Jaccard similarity, where the *Jaccard similarity* between two cells is computed as the number of genes with a signature of 1 in both cells divided by the number of genes with a signatures of 1 in at least one of the cells. *TF-IDF graph-based greedy clustering with Euclidean, Pearson, cosine and Jaccard distances (TF-IDF_Bin_Greedy_E, TF-IDF_Bin_Greedy_P, TF-IDF_Bin_Greedy_C, TF-IDF_Bin_Greedy_J).* In these methods we begin by building an undirected graph with cells as the vertices and edges connecting pairs of cells for which the binarized expression signature vectors have Euclidean, Pearson, cosine, or Jaccard distance below a certain cutoff value. For our experiments we set a rather low cutoff of 0.01 to to build a dense graph, but weighted the edges of this graph by the corresponding pairwise similarity measures for clustering by greedy modularity optimization, which was performed using the algorithm introduced in [21] and implemented in the cluster_fast_greedy() function of the igraph R package [17]. To ensure the homogeneity of resulting clusters and to force a minimum number of clusters when required, all clusters with a silhouette score below a given threshold were subjected to further partitioning. All cells in such a cluster were used to form a new gene expression matrix which was subjected to TF-IDF transformation, binarization, and then clustering via the greedy modularity optimization algorithm. The process was repeated until the minimum number of clusters was achieved, or no cluster had a silhouette score below the given threshold. *TF-IDF graph-based Louvain clustering with Euclidean, Pearson, cosine and Jaccard distances (TF-IDF_Bin_Louvain_E, TF-IDF_Bin_Louvain_P, TF-IDF_Bin_Louvain_C, TF-IDF_Bin_Louvain_J).* Here, the same approach described above for graph-based greedy clustering was used in conjunction with the Louvain modularity optimization algorithm [10] as implemented in the cluster_louvain() function of the igraph R package [17].

### Experimental setup

#### Datasets

**Fig. 5:**
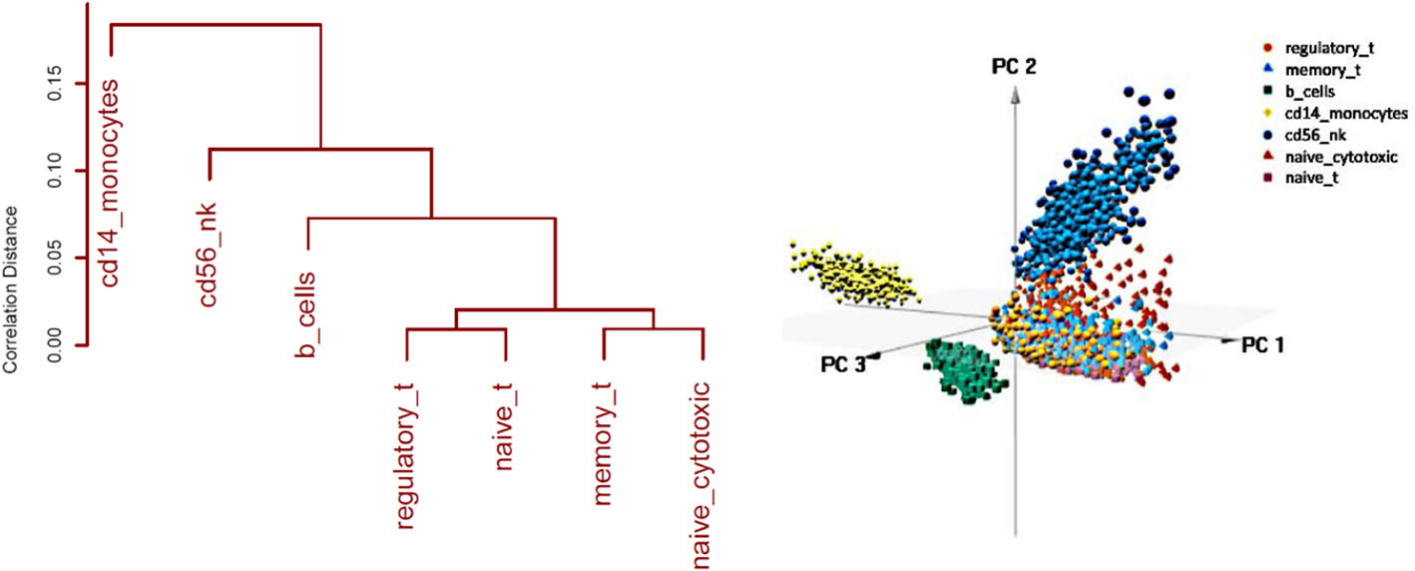
Left: Correlation distances between mean expression levels of 7 immune cell types from [2]. Right: 3D PCA plot of 1000 cells of each type

To assess the accuracy of compared clustering methods we used synthetic mixtures of real scRNA-Seq profiles generated from FACS sorted immune cells using the 10x Genomics platform [2]. We started from the filtered UMI count matrices generated using the CellRanger pipeline and made publicly available at https://support.10xgenomics.com/single-cell-gene-expression/datasets. Of the available sorted cell populations we excluded those shown to have substantial heterogeneity in [2]. This left us with seven cell types: CD4+/CD25+ Regulatory Cells (regulatory_t), CD4+/CD45RO+ Memory Cells (memory_t), CD19+ B Cells (b_cells), CD14+ Monocytes (cd14_monocytes), CD56+ Natural Killer Cells (cd56_nk), CD8+/CD45RA+ Naive Cytotoxic T Cells(naive_cytotoxic), and CD4+/CD45RA+/CD25- Naive T cells (naive_t). The hierarchical clustering dendrogram based on Pearson correlations between mean gene expression levels of the seven cell types along with a 3-dimensional PCA projection of the individual scRNA-Seq profiles are shown in Fig. 5.

Clearly, B cells, NK cells and monocytes are relatively dissimilar to each other and to the four T cell types, which in turn form two highly similar pairs (memory_t and naive_cytotoxic) and (regulatory_t and naive_t) and pairs with intermediate dissimilarity like (memory_t and naive_t) and (regulatory_t and naive_cytotoxic). Thus, in a first set of experiments, we focused on mixtures of cells generated from six pairs of cell types of varying degrees of dissimilarity. We chose pairs (b_cells and cd14_monocytes) and (b_cells and cd56_nk) to represent mixtures of highly dissimilar cell types, pairs (memory_t and naive_cytotoxic) and (regulatory_t and naive_t) to represent mixtures of highly similar cell types, and pairs(memory_t and naive_t) and (regulatory_t and naive_cytotoxic) to represent mixtures of cell types with intermediate similarity. To assess clustering accuracy in the presence of different levels of imbalance between the numbers of cells of different types, for each of the six pairs of cell types we generated mixtures in ratios 7:1, 3:1, 1:1, 1:3, and 1:7. For each mixture ratio, we sampled a total of 1,000 cells from the corresponding cell types. Finally, to assess accuracy on a more complex cell population, we generated mixtures comprised of 7,000 cells sampled from all seven cell types in equal proportions.

**Fig. 6:**
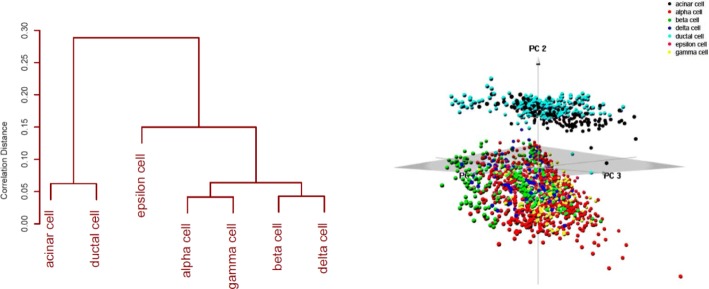
Left: Correlation distances between mean expression levels of 7 pancreatic island cell types from [5]. Right: 3D PCA plot of the 2045 pancreatic island cells

We also tested the implemented methods on scRNA-Seq data from [5]. For this dataset, cells from pancreatic islets were dissociated and sorted by FACS into 384-well plates. Single-cell RNA-seq libraries were generated using the Smart-seq2 protocol and sequenced on an Illumina HiSeq 2000. We used all 2,045 cells annotated with one of seven cell types (185 acinar cells, 886 alpha cells, 270 beta cells, 197 gamma cells, 114 delta cells, 386 ductal cells, and 7 epsilon cells) identified based on known gene markers in [5]. For this dataset we included all cells without any quality filtering to reflect as close as possible the natural frequency of these cell types in pancreatic islets. As in [22], marker genes with unusually high expression levels (INS for beta cells, GCG for alpha cells, SST for delta cells, PPY for PP/gamma cells, and GHRL for epsilon cells) were removed prior to clustering to eliminate the possibility that they drive the clustering by themselves. A hierarchical clustering dendrogram based on the Pearson correlation between mean gene expression levels of the seven cell types and a 3-dimensional PCA projection of the individual scRNA-Seq profiles are shown in Fig. 6.

#### Accuracy measures

For each dataset we computed macro- and micro-accuracy measures [23, 24] defined by:

5 $$ \emph {Micro Accuracy} = {\sum_{i=1}^{K}C_{i}} / {\sum_{i=1}^{K}N_{i}}  $$

6 $$ \emph {Macro Accuracy} = \frac{1}{K} \sum_{i=1}^{K} \frac{C_{i}}{N_{i}}  $$

where *K* is the number of classes, *N*_*i*_ is the number of samples in class *i*, and *C*_*i*_ is the number of correctly labeled samples in class *i*. Note that macro- and micro-accuracy are identical for 1:1 mixtures, but may differ significantly for imbalanced datasets, as macro-averaging gives equal weight to the accuracy of each class (average accuracy of all classes’ accuracies), whereas micro-averaging gives equal weight to each cell classification decision (overall accuracy). The ground truth was based on the cell sorting information and annotations from [2] and [5].

For methods that identified more clusters than expected (more than two clusters for the 2-class experiments or more than seven for the 7-class mixtures), we used majority based matching to label clusters with cell types. For example, if a predicted cluster has *x* cells labeled as cell type C1 in the ground truth and *y* cells labeled as cell type C2, then all cells are assumed to be predicted as cell type C1 for relevant accuracy calculations when *x*>*y*. This approach ensures that methods that are more sensitive to the existing heterogeneity within the true cell types are not penalized as long as the resulting sub-clusters are “pure”, i.e., all or most cells of that sub-cluster belong to only one of the cell types contributing to the mixture. All datasets used in the paper along with a Shiny application that performs accuracy calculations for user uploaded clustering results are available at http://cnv1.engr.uconn.edu:3838/SCA/.

## Results and discussion

**Fig. 7:**
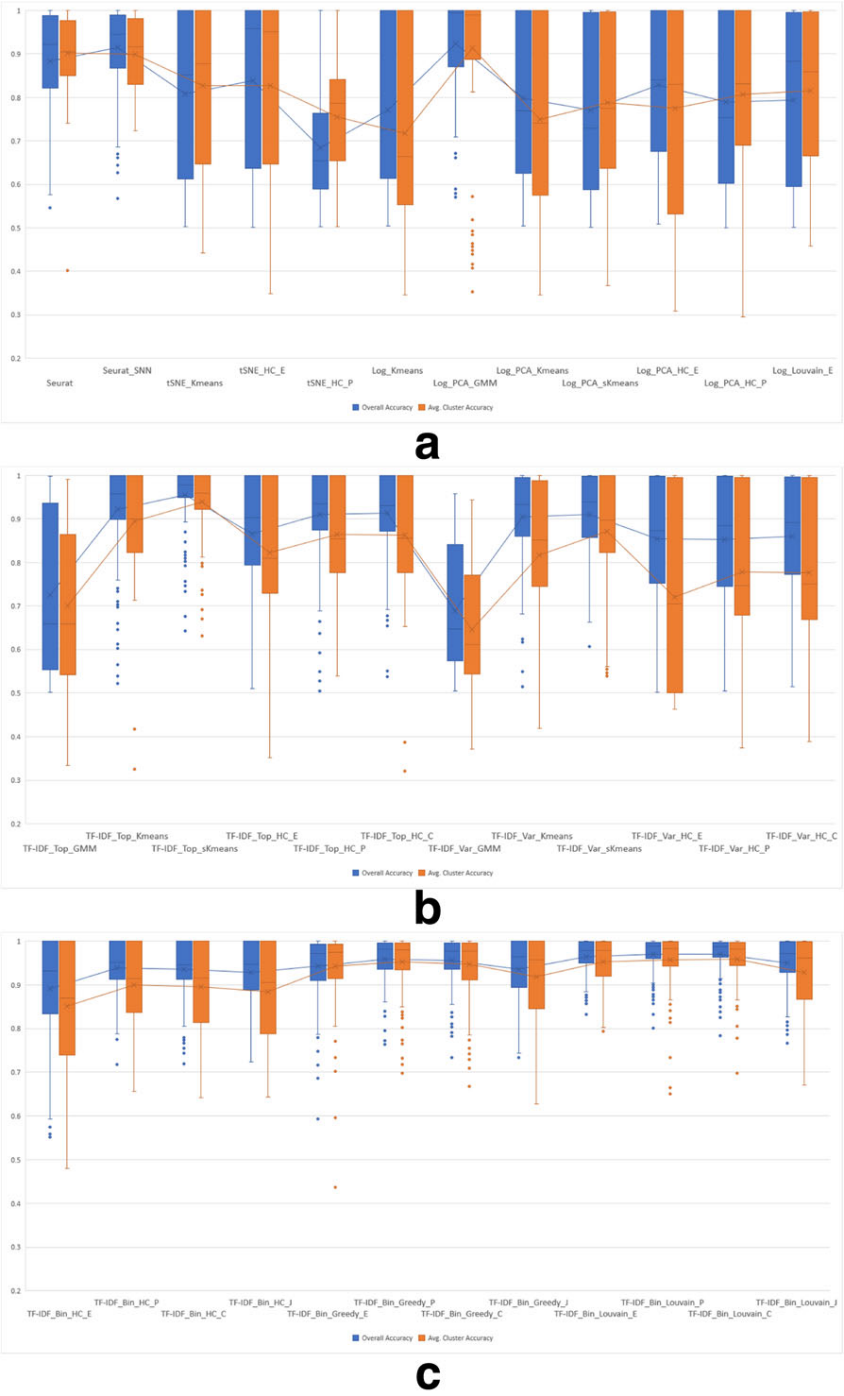
Micro and macro accuracy on 2-class synthetic mixtures of immune cells with ratios 1:1, 1:3/3:1, and 1:7/7:1 for (**a**) existing methods, (**b**) algorithms using TF-IDF based gene selection, and (**c**) algorithms using TF-IDF binarization

Each of the 36 clustering algorithms described in the Methods section was run on 2-class synthetic mixtures of 1,000 cells sampled in different ratios from six pairs of immune cell types as described in Experimental setup. For each combination of cell types and mixture ratio we repeated each experiment five times and computed the macro- and micro-accuracy using Eqs. (5)-(6). Box-and-whiskers plots displaying the macro- and micro-accuracies of the compared algorithms, grouped into three categories (existing methods, algorithms using TF-IDF based gene selection, and algorithms using TF-IDF binarization), are shown in Fig. 7. Each plot shows the median of the corresponding measure as the middle horizontal line, along with mean values as the middle points connected by lines across methods. The whiskers indicate the extreme non-outlier data points of the upper and lower quartiles. If present, outliers, i.e., data points that lie more than 1.5 interquartile ranges below the first quartile or above the third quartile, are indicated as single points on the plot.

Overall, algorithms using TF-IDF binarization have consistently high accuracy in all 2-class experiments, with existing methods and algorithms using TF-IDF based gene selection showing a higher degree of variability in accuracy across datasets. For remaining results we eliminated 8 methods that show consistently lower clustering accuracy in the 2-class experiments. Specifically, from the existing methods group we removed from further analysis tSNE_HC_P, Log_Kmeans, and Log_PCA_sKmeans, all of which have both macro and micro-accuracy averages below 0.8. From the group of methods using TF-IDF based gene selection we removed the two GMM methods (TF-IDF_Top_GMM and TF-IDF_Var_GMM), which clearly performed much worse than the rest. We also removed the three hierarchical clustering methods using genes with highly variable TF-IDF scores (TF-IDF_Var_HC_E, TF-IDF_Var_HC_P, TF-IDF_Var_HC_C) since their accuracy is worse than the corresponding methods that use the genes with top average TF-IDF score. All twelve algorithms using TF-IDF binarization were retained for further in-depth comparisons.

**Fig. 8:**
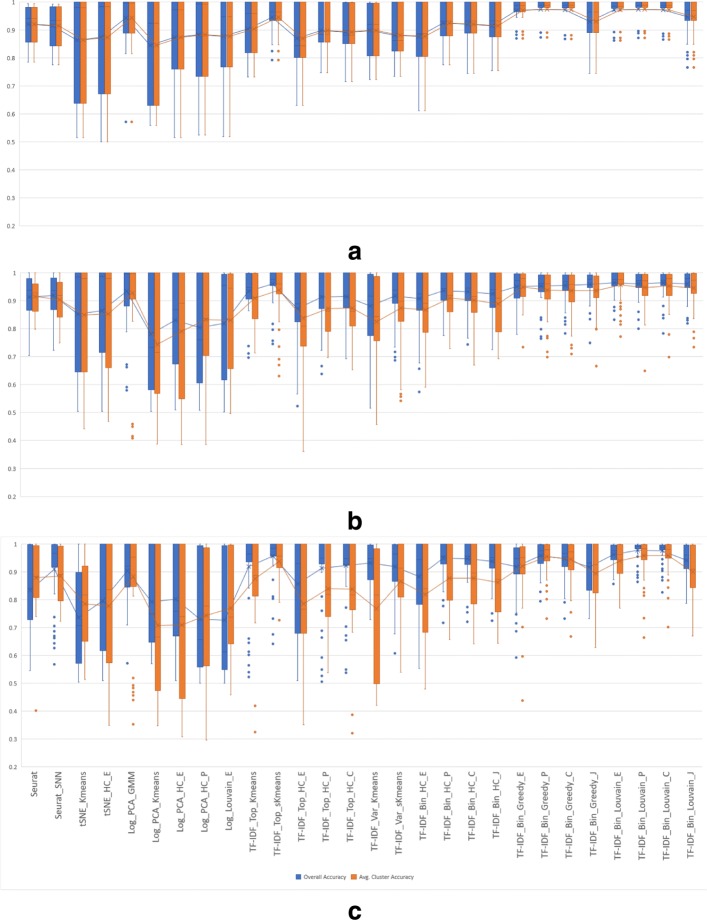
Micro and macro accuracies on 2-class synthetic mixtures with ratios 1:1 (**a**), 1:3/3:1 (**b**), and 1:7/7:1 (**c**)

Box-and-whiskers plots displaying the macro- and micro-accuracies of the 28 remaining algorithms on 2-class synthetic mixtures with varying mixture ratios are shown in Fig. 8. Among existing methods, the Log_PCA_GMM EM-based algorithm and Seurat_SNN have highest average macro and micro-accuracies, with Log_PCA_GMM having an edge in average accuracies on the more balanced 1:1 and 1:3/3:1 mixtures, and Seurat_SNN yielding slightly better macro-accuracy for the more imbalanced 1:7/7:1 mixtures. However, several TF-IDF based clustering methods achieve higher overall average macro- and micro-accuracies for all mixture ratios, with TF-IDF_Bin_Louvain_C, TF-IDF_Bin_Louvain_P and TF-IDF_Top_sKmeans scoring the highest. For imbalanced mixtures, the micro-accuracy is usually lower than but closely tracks macro-accuracy, generally preserving the relative performance of the compared methods.

**Fig. 9:**
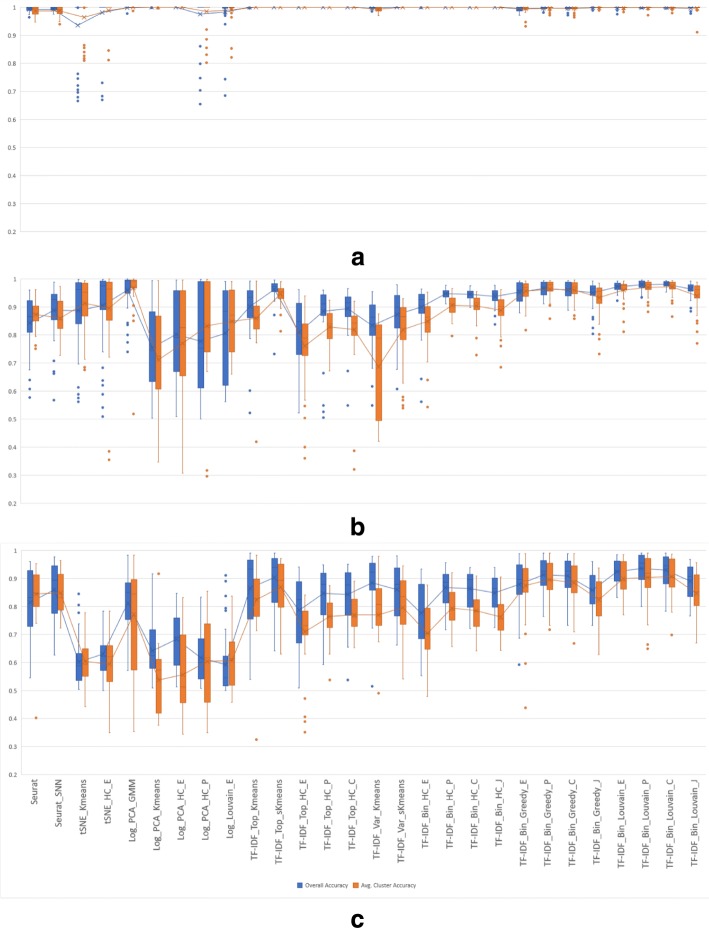
Micro- and macro-accuracies for synthetic mixtures with ratios 1:1, 1:3/3:1, and 1:7/7:1 simulated from (**a**) highly dissimilar cell type pairs (cd14_monocytes,b_cells) and (cd56_nk,b_cells), (**b**) intermediate similarity cell type pairs (regulatory_t,naive_cytotoxic) and (memory_t,naive_t), and (**c**) highly similar cell type pairs (regulatory_t,naive_t) and (memory_t,naive_cytotoxic)

Plots displaying the macro- and micro-accuracies of the 28 methods grouped by the level of similarity of the two cell types in the mixtures are given in Fig. 9. As expected, all methods have very high clustering accuracy on mixtures of highly dissimilar cell types. The accuracy is generally lower on mixtures of cell types with intermediate similarity, and lower still on mixtures of highly similar cell types. Algorithms based on TF-IDF binarization perform among the best on all types of mixtures, with TF-IDF_Bin_Louvain_C and TF-IDF_Bin_Louvain_P showing most consistent performance. The TF-IDF_Top_sKmeans algorithm is best-performing within the group of algorithms using TF-IDF based gene selection, with only slightly lower performance than TF-IDF_Bin_Louvain_C and TF-IDF_Bin_Louvain_P on mixtures of highly similar pairs.

**Fig. 10:**
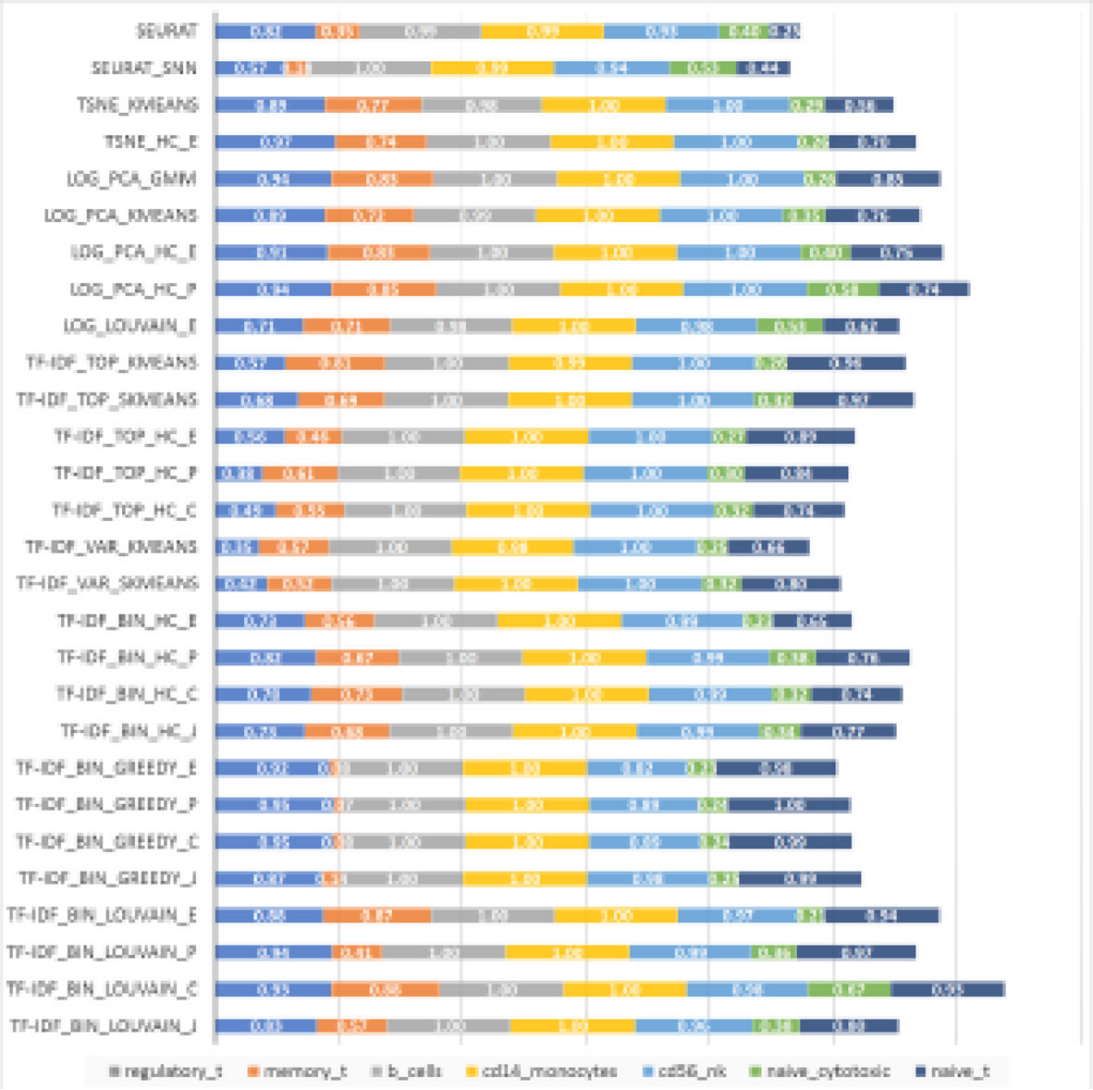
Accuracy for equal-proportion 7-way mixtures of immune cell types (1000 cells each)

To assess the effect of increased population complexity on accuracy, we also ran the 28 methods on equal-proportion mixtures consisting of all seven immune cell types from [2]. The accuracies achieved for each cell type are shown in Fig. 10. Since the cell types were mixed in equal proportions in this experiment, the macro- and micro-accuracy of each method are equal to the average accuracy over all cell types, and hence proportional to the total length of the horizontal bars in the figure. These mixtures contain both highly similar and highly dissimilar cell types, and several methods end up assigning highly similar cell types to a single cluster, resulting in significantly reduced accuracy for some of the cell types. TF-IDF_Bin_Louvain_C is least affected by such miss-assignments, achieving the best overall accuracy.

**Fig. 11:**
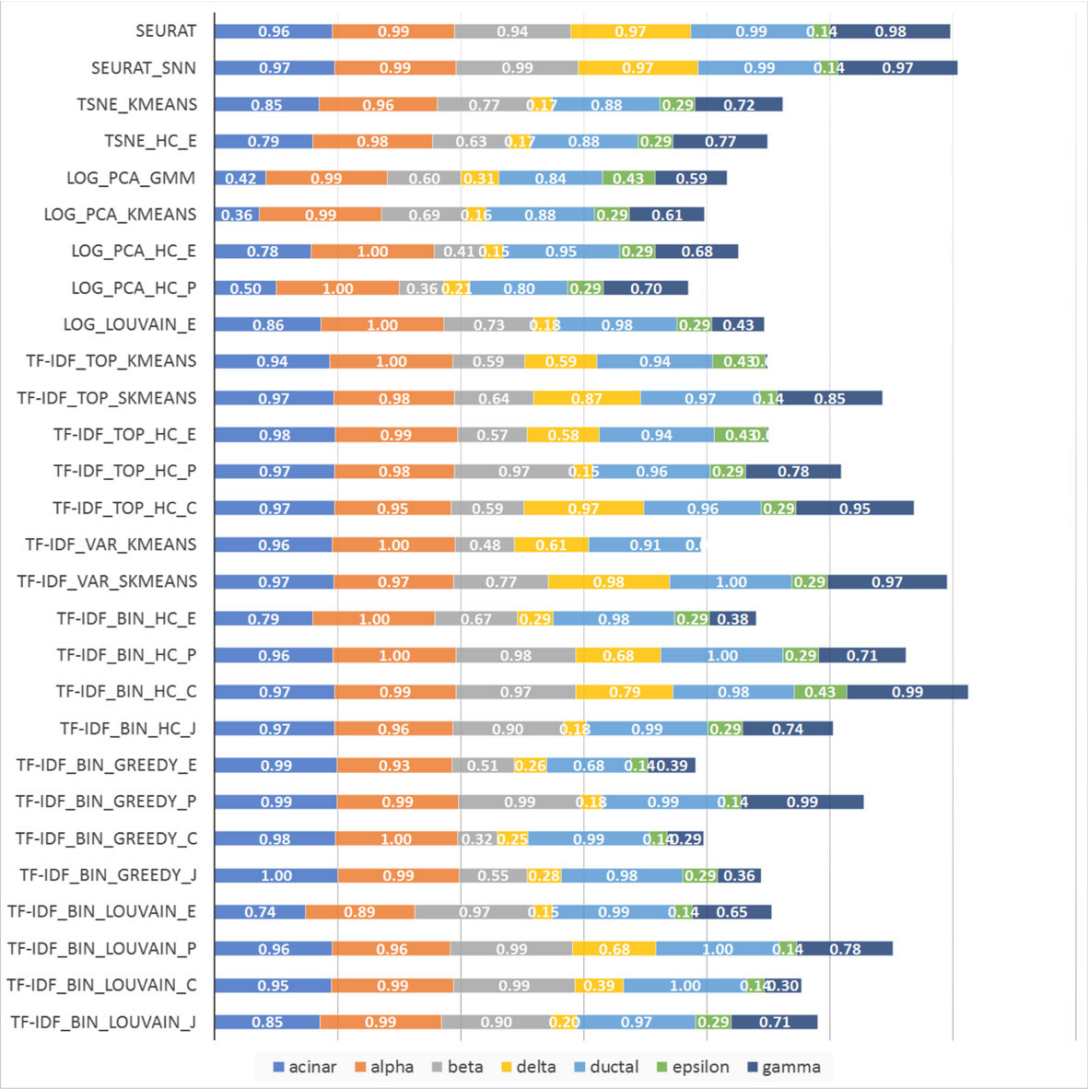
Accuracy for pancreatic cells based on raw counts

**Fig. 12:**
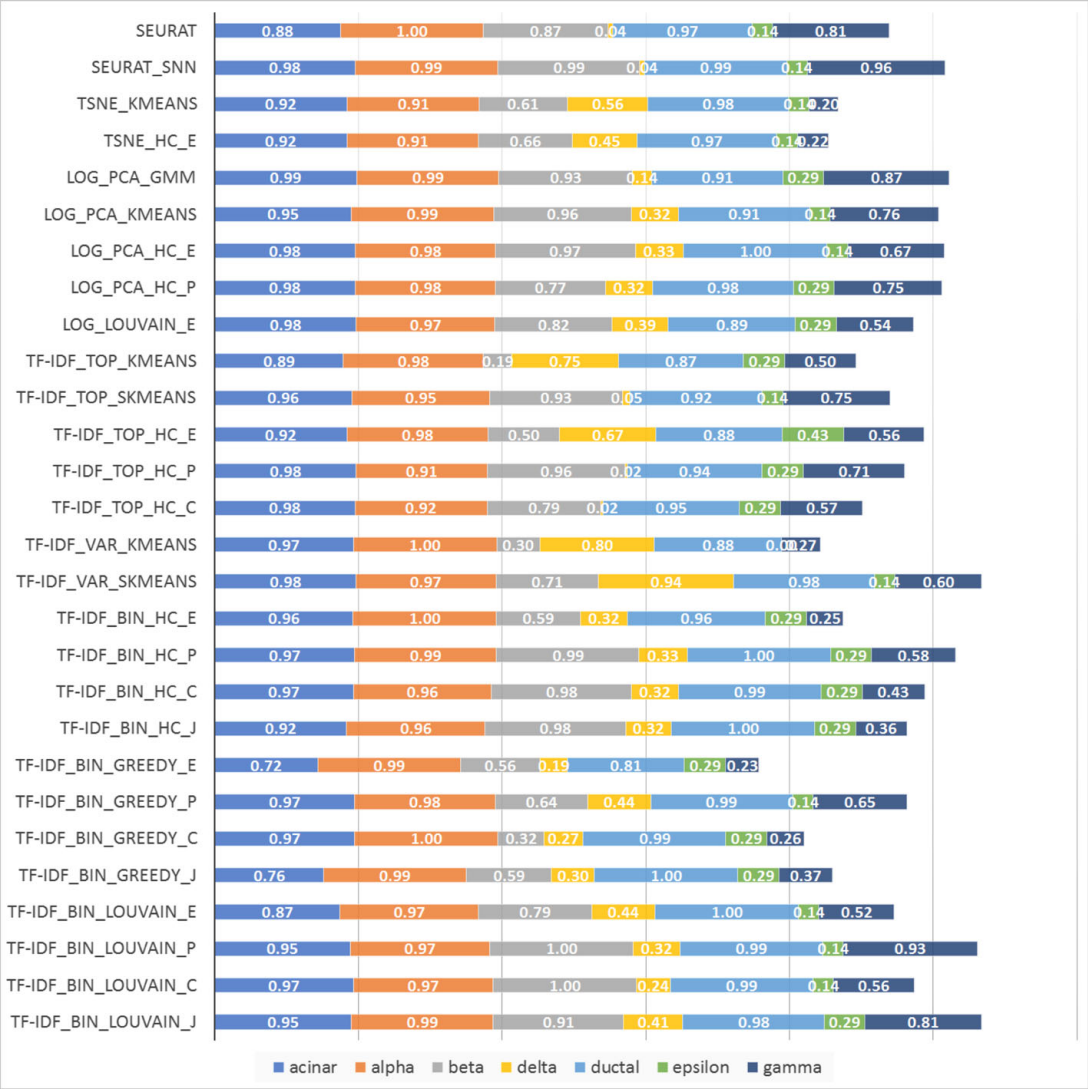
Accuracy for pancreatic cells based on the RPKM values

Figures 11 and 12 show the accuracy per cell type for experiments on the scRNA-Seq dataset from [5], consisting of 2,045 pancreatic islet cells annotated with one of seven cell types. Since cell type abundances in this dataset reflect their natural frequency in pancreatic islets, the total length of the horizontal bars in the figure is proportional with the macro-accuracy (but not necessarily micro-accuracy) of each method. Two sets of results are presented, one based on raw counts and one based on RPKM values in [5]. The relative performance of the compared methods on this dataset is quite different from that on the 7-way mixture in Fig. 10, underscoring the fact that the performance of clustering algorithms is highly dependent on specific aspects of each dataset. The relative performance is also dependent on the metric used, with raw counts yielding a quite different ranking of methods compared to RPKMs.

Tables 1 and 2 summarizes the results of all experiments by giving the average rank (among the 28 selected methods) achieved on each dataset based on macro-, respectively micro-accuracy, along with overall rank averages that give equal weight to each dataset. TF-IDF_Bin_Louvain_C has the lowest overall average rank with respect to both macro- and micro-accuracy. The next three best performers with respect to overall average rank for both macro- and micro-accuracy are all based on the TF-IDF transform as well (in order, TF-IDF_Bin_Louvain_P, TF-IDF_Bin_Louvain_E, and TF-IDF_Top_sKmeans), with TF-IDF_Bin_Greedy_P coming fifth in macro-accuracy overall average rank (Log_PCA_GMM takes fifth place for micro-accuracy average rank).

**Table 1:** Average ranks based on micro-accuracy

Methods	M Nc	R N	M N	R Nc	B Nk	B Mc	7-class	Pancreas	Avg.
Seurat	14.6	19.0	25.0	25.6	**1.0**	25.6	28.0	**4.0**	17.9
Seurat_SNN	6.8	13.8	21.0	18.4	**1.0**	25.6	26.6	**1.0**	14.3
tSNE_Kmeans	26.0	27.0	14.6	18.6	22.6	27.8	11.4	19.5	20.9
tSNE_HC_E	25.0	25.4	12.6	18.0	6.0	11.2	10.0	20.0	16.0
Log_PCA_GMM	20.8	10.6	**2.4**	12.8	**1.0**	**1.0**	**4.4**	14.5	8.4
Log_PCA_Kmeans	24.4	24.4	26.4	26.8	**1.0**	**1.0**	7.6	14.0	15.7
Log_PCA_HC_E	23.8	22.8	22.6	23.8	**1.0**	**1.0**	**4.6**	14.0	14.2
Log_PCA_HC_P	27.0	25.2	25.4	26.0	16.4	6.0	**2.4**	18.5	18.4
Log_Louvain_E	26.2	27.2	25.8	21.0	15.4	6.2	10.4	14.0	18.3
TF-IDF_Top_Kmeans	6.0	16.8	15.8	17.0	**1.0**	**1.0**	9.2	21.0	11.0
TF-IDF_Top_sKmeans	**2.0**	7.4	7.0	2.4	**1.0**	**1.0**	8.4	9.5	**4.8**
TF-IDF_Top_HC_E	20.4	21.0	24.4	23.4	**1.0**	**1.0**	19.8	18.5	16.2
TF-IDF_Top_HC_P	14.8	15.8	19.2	16.0	**1.0**	**1.0**	16.4	12.0	12.0
TF-IDF_Top_HC_C	14.6	17.0	17.4	15.4	**1.0**	**1.0**	18.0	14.5	12.4
TF-IDF_Var_Kmeans	7.2	10.6	19.0	24.2	10.0	**1.0**	25.8	21.5	15.0
TF-IDF_Var_sKmeans	11.0	15.2	19.4	18.2	**1.0**	**1.0**	20.2	**4.5**	11.3
TF-IDF_Bin_HC_E	21.0	21.4	17.4	14.6	**1.0**	**1.0**	17.0	19.5	14.1
TF-IDF_Bin_HC_P	13.6	9.4	8.4	9.2	**1.0**	**1.0**	8.0	**6.0**	7.1
TF-IDF_Bin_HC_C	14.0	10.8	11.4	9.2	**1.0**	**1.0**	10.6	8.5	8.3
TF-IDF_Bin_HC_J	17.4	13.2	13.4	9.8	**1.0**	**1.0**	12.8	14.0	10.3
TF-IDF_Bin_Greedy_E	11.6	7.4	7.2	8.8	18.8	5.8	23.8	27.0	13.8
TF-IDF_Bin_Greedy_P	**4.6**	**4.6**	**5.2**	2.4	**5.0**	**1.0**	19.0	12.0	**6.7**
TF-IDF_Bin_Greedy_C	**5.2**	**5.2**	7.8	**2.8**	23.2	**1.0**	19.4	28.0	11.6
TF-IDF_Bin_Greedy_J	16.2	9.4	10.6	6.4	5.8	**1.0**	18.0	24.5	11.5
TF-IDF_Bin_Louvain_E	5.8	**2.0**	**3.2**	**2.4**	**5.0**	**1.0**	**4.2**	13.0	**4.6**
TF-IDF_Bin_Louvain_P	**1.0**	**1.4**	**1.8**	**1.4**	**1.0**	**1.0**	14.2	**4.0**	**3.2**
TF-IDF_Bin_Louvain_C	**1.2**	**2.0**	**1.6**	**1.0**	**1.0**	**1.0**	**1.2**	11.5	*2.6*
TF-IDF_Bin_Louvain_J	9.6	6.2	6.0	**2.8**	18.4	**1.0**	11.8	7.0	7.9

The lowest five average ranks (including ties) for each dataset are typeset in bold, and the best overall average rank is shown in italic

**Table 2:** Average ranks based on macro-accuracy

Methods	M Nc	R N	M N	R Nc	B Nk	B Mc	7-class	Pancreas	Avg.
Seurat	8.2	8.0	18.8	24.2	**1.0**	26.4	27.2	10.0	15.5
Seurat_SNN	9.0	9.2	18.0	19.4	**1.0**	27.0	27.0	**3.5**	14.3
tSNE_Kmeans	24.2	24.0	9.0	14.8	22.4	26.6	11.6	18.5	18.9
tSNE_HC_E	24.4	24.8	9.4	18.2	6.2	10.4	9.2	20.0	15.3
Log_PCA_GMM	20.4	6.4	**3.0**	**4.8**	**1.0**	**1.0**	**4.4**	14.0	**6.9**
Log_PCA_Kmeans	27.2	27.4	26.6	26.8	**1.0**	**1.0**	7.6	16.0	16.7
Log_PCA_HC_E	27.2	24.8	22.0	24.6	**1.0**	**1.0**	**4.8**	13.5	14.9
Log_PCA_HC_P	25.8	23.8	17.8	20.6	16.2	5.6	**2.4**	18.0	16.3
Log_Louvain_E	23.0	25.6	20.8	14.2	15.0	5.8	10.6	14.5	16.2
TF-IDF_Top_Kmeans	9.6	13.4	18.6	13.6	**1.0**	**1.0**	9.6	18.5	10.7
TF-IDF_Top_sKmeans	**4.2**	9.4	8.0	6.2	**1.0**	**1.0**	8.6	12.5	**6.4**
TF-IDF_Top_HC_E	21.4	19.2	25.6	23.6	**1.0**	**1.0**	17.4	13.0	15.3
TF-IDF_Top_HC_P	17.6	17.4	20.4	20.6	**1.0**	**1.0**	18.2	11.5	13.5
TF-IDF_Top_HC_C	17.0	16.2	20.6	21.2	**1.0**	**1.0**	20.4	12.5	13.7
TF-IDF_Var_Kmeans	12.0	21.0	27.4	27.6	19.6	19.0	26.4	24.0	22.1
TF-IDF_Var_sKmeans	11.8	18.0	23.4	18.6	**1.0**	**1.0**	21.6	**2.5**	12.2
TF-IDF_Bin_HC_E	20.2	22.2	19.8	16.6	**1.0**	**1.0**	19.2	21.0	15.1
TF-IDF_Bin_HC_P	15.4	13.2	12.0	12.2	**1.0**	**1.0**	8.4	**4.5**	8.5
TF-IDF_Bin_HC_C	15.8	15.2	13.2	11.4	**1.0**	**1.0**	10.8	**5.5**	9.2
TF-IDF_Bin_HC_J	17.8	15.8	14.0	12.8	**1.0**	**1.0**	13.0	12.0	10.9
TF-IDF_Bin_Greedy_E	7.0	**5.2**	5.4	**4.8**	20.0	**1.0**	23.0	27.0	11.7
TF-IDF_Bin_Greedy_P	**3.8**	**4.2**	**4.4**	**2.2**	**1.0**	9.8	19.2	11.0	7.0
TF-IDF_Bin_Greedy_C	4.8	**5.0**	5.6	**3.2**	**1.0**	9.8	19.4	26.5	9.4
TF-IDF_Bin_Greedy_J	13.8	10.2	11.0	6.2	10.2	**1.0**	16.2	22.0	11.3
TF-IDF_Bin_Louvain_E	**4.4**	**2.6**	**3.4**	6.4	**1.0**	**1.0**	**4.2**	16.0	**4.9**
TF-IDF_Bin_Louvain_P	**1.2**	**3.4**	**2.4**	**2.4**	10.0	**1.0**	11.2	**4.0**	**4.5**
TF-IDF_Bin_Louvain_C	**1.0**	**3.0**	**1.8**	**2.4**	**5.2**	**1.0**	**1.2**	11.5	*3.4*
TF-IDF_Bin_Louvain_J	9.4	8.2	9.8	5.4	5.6	**1.0**	12.0	6.5	7.2

The lowest five average ranks (including ties) for each dataset are typeset in bold, and the best overall average rank is shown in italic

## Conclusions

In this paper we compared eight methods for clustering scRNA-seq data: nine commonly used existing approaches and nineteen methods based on the use of TF-IDF scores similar to those used in the text analysis field. Empirical experiments on a variety of cell types and ratio mixtures suggest that TF-IDF based methods achieve consistently high accuracy, even on complex mixtures of highly similar cell types.

A limitation of the TF-IDF_Bin_HC methods’ group is the quadratic time required for distance calculations used in hierarchical clustering methods, which becomes a performance bottleneck for datasets with millions of single cells. In ongoing work we are exploring MinHashing [25, 26] and Locality Sensitive Hashing (LSH) [27–30] techniques that make feasible the efficient computation of highly similar pairs of cells under, e.g., Jaccard or cosine distances [31, 32].
